# Effectiveness of the “What’s Up!” Intervention to Reduce Stigma and Psychometric Properties of the Youth Program Questionnaire (YPQ): Results from a Cluster Non-randomized Controlled Trial Conducted in Catalan High Schools

**DOI:** 10.3389/fpsyg.2017.01608

**Published:** 2017-09-14

**Authors:** Laura Andrés-Rodríguez, Adrián Pérez-Aranda, Albert Feliu-Soler, María Rubio-Valera, Ignacio Aznar-Lou, Antoni Serrano-Blanco, Miquel Juncosa, Anaïs Tosas, Albert Bernadàs, Juan V. Luciano

**Affiliations:** ^1^Institut de Recerca Sant Joan de Déu Barcelona, Spain; ^2^Teaching, Research & Innovation Unit, Parc Sanitari Sant Joan de Déu Barcelona, Spain; ^3^Primary Care Prevention and Health Promotion Research Network (RedIAPP) Madrid, Spain; ^4^Centre for Biomedical Research in Epidemiology and Public Health Madrid, Spain; ^5^OBERTAMENT Initiative Barcelona, Spain

**Keywords:** stigma, mental disorders, non-randomized controlled trial, Youth Program Questionnaire, Reported and Intended Behavior Scale

## Abstract

Mental disorders are highly prevalent in the general population, and people who experience them are frequently stigmatized. Stigma has a very negative impact on social, academic/professional, and personal life. Considering the high rates of mental disorders among children and adolescents (13.4%) and how critical this age is in the formation of nuclear beliefs, many campaigns to combat stigma have been developed in the last decade, with mixed results. The OBERTAMENT initiative has produced various anti-stigma campaigns in Catalonia (Spain). In the present study, the main objective was to report on the effectiveness of the OBERTAMENT “*What’s up!*” intervention, a curricular intervention including education and social contact conducted by the teachers in the classroom with teenagers aged between 14 and 18. Prior to this, we examined the psychometric properties of the Youth Program Questionnaire (YPQ), our main outcome measure, in terms of dimensionality, reliability, and validity. A cluster non-randomized controlled trial was conducted to assess this intervention, which was tested in nine high schools situated in the Barcelona region. A convenience sample of 261 students formed the intervention group and 132 the control group (52% women, mean age = 14, *SD* = 0.47). The assignment to study conditions was conducted by Departament d’Ensenyament (Department of Education), Generalitat de Catalunya (Catalan Government). Participants were evaluated at baseline, post-intervention, and 9-month follow-up. The main outcome measure of this study was the YPQ. The Reported and Intended Behavior Scale (RIBS) was used as secondary outcome measure. The statistical analysis indicated that the YPQ possesses a two-factor structure (stereotypical attitudes and intended behavior) and sound psychometric properties. The multilevel mixed-effects models revealed statistically significant interactions for both study measures and *post hoc* intragroup analyses revealed a significant but small improvement in the YPQ and RIBS scores in the intervention group. Overall, our results indicate that “*What’s up!*” produced statistically significant, albeit small improvements in stereotypical attributions and intended behavior toward people with mental disorders. Some methodological limitations and the relatively low levels of stigma observed in our sample may undermine our results. The implications of our results are discussed in relation to stigma research.

## Introduction

Mental disorders have a high prevalence in the general population. According to the most recent evidence, 38.2% of the general European population experiences at least one mental disorder, which corresponds to an estimated 164.7 million people (Wittchen et al., 2011). In Spain, the European Study of the Epidemiology of Mental Disorders (ESEMeD; Haro et al., 2003) concluded that approximately one out of every five people who were surveyed had presented a mental disorder at some point of their life, and 8.5% of the population had experienced a mental disorder in the previous year. Moreover, children and teenagers are a high-risk population since a worldwide prevalence of 13.4% for any mental disorder has been recently reported (Polanczyk et al., 2015).

It is noteworthy, that people experiencing mental disorders frequently deal with stigma associated with their condition (Hugo et al., 2003; Ando et al., 2013; Loch et al., 2014). Stigma is a multidimensional phenomenon that includes cognitive and behavioral aspects (Corrigan and Shapiro, 2010). The first one is, in turn, formed out of two constructs: *stereotypes*, which are related to knowledge (Thornicroft et al., 2007); and *prejudices*, which are the generalized attitudes toward members of a social group. These cognitive aspects of stigma begin to form and consolidate during adolescence (Flavell et al., 2002; Schulze et al., 2003) and their consequence is *discrimination*, the behavioral aspect of stigma. In the case of mental disorders, a common example would be an employer who, based on the belief that people with a mental disorder are violent (stereotype), has a negative feeling -fear, anxiety- toward them (prejudice) and, thus, decides not to hire a person who experiences one (discrimination) (Crespo et al., 2008; Ke et al., 2015).

Stigma has many consequences: it contributes to low self-esteem and quality of life (Livingston and Boyd, 2010) and has a negative effect on adequate housing, work and financial status (Rüsch et al., 2005; Sharac et al., 2010); from a clinical perspective, it has a negative influence on symptom severity and compliance with treatment (Li et al., 2014) and may trigger suicidal ideation and behavior (Rüsch et al., 2014). When stigma is internalized (i.e., self-stigma), it is associated with rejection of help, avoidance of treatment and limited prospects of recovery, among other damaging consequences (Hing and Russell, 2017). These are some reasons why stigma has been identified as one of the greatest challenges facing mental health (Hing and Russell, 2017).

In Spain, the level of stigma toward mental disorders is in line with or slightly lower than in other European countries, with mean Reported and Intended Behavior Scale (RIBS; Evans-Lacko et al., 2011) score corresponding to the 76th percentile (Aznar-Lou et al., 2016), which indicates relatively low levels of stigma. However, this does not mean that the Spanish population should not be subjects of anti-stigma interventions as, for instance, knowledge of mental disorders has been found to be low. A study conducted by López-Ibor concluded that 83% of the surveyed Spanish population knew nothing about schizophrenia and 33% did not know about the origin and causes of the disorder even though 44% affirmed that schizophrenia is not a curable illness. In other countries, such as the United Kingdom, it was reported that the general population, and youngsters particularly, had many misconceptions about mental disorders (Crisp et al., 2000). Similarly, Crespo et al. (2008) reported a moderate degree of knowledge about the treatment of mental disorders, the work options for people who experience them, and the causes and degree of awareness of the disorder in a general population sample.

Nonetheless, knowledge about mental disorders is not the only aspect to be improved regarding stigma, as there is evidence that public attitudes have not changed over the last two decades, or have even become worse regarding people with psychotic disorders (Hansson and Markström, 2014). Some studies have identified several personal characteristics that are associated with having a higher degree of stigma toward people who experience mental disorders, such as being male (Stickney et al., 2012), having had no previous contact with people with mental conditions (Batterham et al., 2013), lower educational levels and living alone (Coppens et al., 2013). In Spain, some factors such as having completed secondary or university education and having had contact with people with mental problems are related to a better attitude toward mental disorders and more favorable intended behavior (Aznar-Lou et al., 2016).

Bearing this in mind, various approaches have attempted to reduce stigma toward mental disorders through education and social contact. Corrigan and Penn (1999) considered educational interventions to be one of the most effective ways of reducing stigma. Educational approaches often challenge inaccurate stereotypes of mental disorders, replacing them with factual information (Corrigan and Shapiro, 2010; Economou et al., 2012). These interventions are frequently addressed to children and teenagers as they constitute one of the most indicated populations for many reasons: studies have proved that children do not yet have a consolidated idea of what mental illness denotes (Corrigan and Watson, 2007), and the personality traits that constitute the foundation for stereotype endorsement are not well-entrenched until adolescence (Flavell et al., 2002), considering that this stage lasts, approximately, from 13 to 18 years old. Besides, as previously mentioned, adolescents form a population which is severely affected by mental disorders, in part due to the great challenges that this life stage entails (e.g., identity definition, sexual role and body changes, academic goals).

Therefore, interventions targeted at these age groups seem to be especially useful, not only with preventive aims but also to combat the stigma that children and adolescents already begin to experience and that frequently damages their social and academic development. Some countries, such as England, have prioritized the topic of improving mental health knowledge and reducing stigma in schools and colleges. In this case, the Public Health England and the Children & Young People’s Mental Health Coalition produced the Promoting Children and Young People’s Emotional Health and Wellbeing (2015) guide, with key evidence-based guidelines for teachers and college principals to create a whole-school approach to promoting emotional health and well-being. This approach does not simply involve a workshop on mental health, but a series of regular practices to be carried out in every school space. By using this model, it may be possible to overcome the limitations of the short-duration educational interventions that have been commonly used until date, which have generally been related to small and short-term improvements in attitudes toward mental disorders in adolescents (Bulanda et al., 2014). These interventions’ effects have been reported to last over 1–6 months (Pinfold et al., 2003; Schulze et al., 2003), and regarding its content, it has been assessed that adding social contact can either imply lower effects for adolescents (Corrigan et al., 2012) or no difference at all in comparison to education alone (Schulze et al., 2003; Warner, 2005; Yamaguchi et al., 2011).

The evaluation of the effectiveness of educational interventions is generally carried out by self-reported measures that evaluate both the cognitive and behavioral components of stigma. Many different measures have been developed, but most present some methodological issues which question their adequacy, and there is no consensus on which one to use (Sakellari et al., 2011). Furthermore, validations of stigma measures oriented to teenagers are scarce worldwide (Ochoa et al., 2015). In this regard, the Youth Program Questionnaire (YPQ), an instrument developed for the campaign “*Opening Minds”* which was conducted in Canada in 2013 (Stuart et al., 2014), is considered to be a good instrument as it measures both the cognitive and behavioral aspects of stigma and is addressed specifically to adolescents. Nonetheless, its psychometric properties have yet to be established.

In Catalonia, the OBERTAMENT initiative, created in 2010, has developed various campaigns to fight stigma associated with mental disorders. The present study has the main objective of evaluating the effectiveness of *“What’s up!”* on reducing perceived stigma in a Catalan sample of adolescent students using the self-report YPQ as primary outcome and the RIBS (Evans-Lacko et al., 2011) as a secondary outcome. *“What’s up!”* is a curricular intervention which includes both education and social contact, created by the OBERTAMENT initiative and addressed to 14–18 year-old Catalan students to reduce stigma levels. Since the YPQ has not yet been validated, its psychometric properties in our Catalan adolescent sample are also reported.

## Materials and Methods

### Study Design

This is a cluster non-randomized controlled trial designed to assess the intervention “*What’s up!*” in nine high schools in the region of Barcelona (Spain) with pre–post and follow up evaluations.

This study was performed in accordance with ethical standards established in the 1964 Declaration of Helsinki and its subsequent updates and established in the World Psychiatric Association Declaration of Madrid. The study protocol was approved by the ethics committee at the Sant Joan de Déu Foundation (CEIC PIC-107-14; Esplugues de Llobregat, Spain). We report this non-randomized controlled trial following Transparent Reporting of Evaluations with Non-randomized Designs guidelines (TREND; Des Jarlais et al., 2004).

### Participants

A total of 446 students aged between 14 and 18 years were recruited from nine different high schools: five (Pere Vives i Vich, Montbui, Salvador Claramunt, Escola Pia, and Maristes) were assigned to the intervention group, and four (Pla de les Moreres, Escola Anoia, IES Joan Mercader, and IES Guinovarda) to the control group. There were no other inclusion/exclusion criteria for participation in this study. All centers were located in the Barcelona region. These high schools were selected by convenience by the Catalan Government (Generalitat de Catalunya) Department of Education. This public entity performed the allocation to study conditions and matched high schools considering the number of students and whether they were private (Escola Pia, Maristes and Escola Anoia) or public centers (Pere Vives i Vich, Montbui, Salvador Claramunt, Pla de les Moreres, IES Joan Mercader and IES Guinovarda). All nine high schools are coeducational and have a maximum of 25 students per class.

### Intervention

The “*What’s up!”* intervention consists of a multifocal action to combat stigma from the classroom. The teachers were given a manual with nine didactic units which contained information, examples, and exercises about mental health problems, and they were in charge of using these units in their classes, with the freedom to choose how and when to do so. This intervention model was conceived to make “*What’s up!”* truly implementable in the real context of high schools. It was indicated that every unit had to be implemented for 1 week. The materials that were selected for each unit were chosen by the OBERTAMENT team and based on evidence from previous effective interventions. The protocol explained in detail different types of exercises that the students should do both individually and in groups for every didactic unit. The teachers were given instructions to employ at least three of the nine didactic units, with the first and last considered to be “essential.”

The nine didactic units corresponded to different school subjects: Language (Catalan and Spanish), Foreign Language (English and French), Sciences (Biology and Geology), Mathematics, Physical Education, and Culture and Ethical Values. It was in this last unit that a person with a mental disorder reported firsthand experiences to the students in the classroom, providing the social contact component of the intervention. **Table 1** shows which units were applied in each high school.

**Table 1:** Units of the curricular intervention and their implementation in every high school.

Unit	Escola Pia (*n* = 90)	Institut Montbuí (*n* = 54)	Maristes Igualada (*n* = 54)	Escola Pere Vives (*n* = 24)	Salvador Claramunt (*n* = 57)
*The standup kid* (English)	One week	One week	One week	One week	One week
*I’m Michael* (English)	One week	–	–	–	One week
*Donne lui la parole* (French)	–	One week	One week	–	–
*What do you know?* (Catalan)	One week	–	One week	Partially implemented	One week
*Health dimension* (Biology)	One week	–	One week	One week	One week
*What do we now about health?* (Maths)	One week	One week	One week	One week	One week
*Activate your well-being* (P.E.)	One week	One week	Partially implemented	–	–
*Let’s fight stigma* (Culture and Ethic Values)	One week	One week	One week	One week	One week
*Testimonials* (Culture and Ethic Values)	One week	One week	One week	One week	One week

*Partially implemented means that not all the materials of the didactic unit where applied.*

Although the teachers did not receive any specific training from OBERTAMENT on how to provide support on mental health disorders, general knowledge about what mental disorders are and how to manage classrooms with heterogeneous needs are part of every teacher’s regular academic training.

Further information on this project is available here (in Spanish): https://obertament.org/es/educacion/proyecto-what-s-up.

### Procedure

After an initial meeting with the education department and all the heads of the high schools, the *“What’s up!”* project team presented the curricular project protocol and the study goals to the teachers who would implement it.

Data from study participants were obtained at three different times: before the intervention, after the intervention (1–3 months, depending on the high school), and at follow-up 9 months from study commencement. Students were aware of their participation in a study but they did not know to which condition their high school was allocated. However, teachers administering the interventions and those assessing outcomes were not blind to study allocation. All three evaluations were conducted in the participants’ classrooms using a paper and pencil self-administered questionnaire. As they were not adults, informed consent was signed by their parents or legal guardians prior to the first evaluation. The same version of the battery of measures was administered in all high schools.

The YPQ was translated from English into Catalan by two native bilingual English/Catalan speakers. Any discrepancies between the Catalan and English versions were resolved by agreement.

### Measures

To evaluate previous contact and experience with people with a mental disorder, four ad hoc questions in dichotomous format (yes/no) were included, along with a further question regarding personal experiences of having a mental disorder.

• The main outcome measure was the YPQ adapted to Catalan, an instrument developed by the Canadian initiative *“Opening minds”* (Stuart et al., 2014) which has two 11-item scales; the Stereotype Scale (YPQ-SS) which measures stereotypic attributions (controllability of the illness, potential for recovery, and potential for violence and unpredictability) and the Social Acceptance Scale (YPQ-SAS) which measures behavioral intentions related to social acceptance. The YPQ is rated on a five-point Likert scale (from 1 to 5) where lower scores indicate lower levels of stigma toward people with a mental disorder. Items 14, 16, 19, 20, 21, and 22 are reverse scored. This study uses the total mean and subscales scores. The psychometric characteristics of this questionnaire are described below. Two exemplary items of this questionnaire are “People with mental illnesses need to be locked away” (YPQ-SS) and “If I knew someone had a mental illness I would not date them” (YPQ-SAS). All the items can be found in the table added as Supplementary Material.
• The Catalan version of the RIBS (Evans-Lacko et al., 2011) was also used to measure intended behavior in relation to future contact with people with a mental disorder (intention to live with, work with, live nearby and continue a relationship with someone with a mental disorder). The four items are rated on a five-point Likert scale from 1 (strongly agree) to 5 (strongly disagree) so that lower scores indicate more favorable intended behavior. In the present study, we use the mean item score. In this project, internal consistency (Cronbach’s α) of RIBS was 0.81, indicating adequate reliability (α ≥ 0.8 is defined as “good”; Nunnally, 1978). An example of an item in this scale is “In the future, I would be willing to live nearby to someone with a mental health problem.”

### Data Analyses

Student’s *t*-test for continuous values and χ^2^ tests with continuity corrections for categorical values were computed on baseline measures and socio-demographic variables to examine differences between the two groups.

Firstly, to analyze the effectiveness of an intervention, we need outcome measures that are well-designed and have empirically proven their reliability and validity. Therefore, we analyzed the psychometric properties of the YPQ in terms of factor structure, internal consistency, and construct validity. To this end, CFA was conducted to test the fit of the two-factor model proposed by the Mental Health Commission of Canada (MHCC; Stuart et al., 2014). In addition, we tested a two-factor model with method effects (model 2 as respecification of model 1) that incorporated correlated error terms on the negatively phrased items. The weighted least squares mean and variance (WLSMV) was used as an estimation method to test the fit of the factor models. The following indices were examined to evaluate model fit (Schumacker and Lomax, 2010): chi-square (non-significance reflects good fit), the Tucker-Lewis index (TLI ≥ 0.9), the comparative fit index (CFI ≥ 0.9), and the root means square error of approximation (RMSEA ≤ 0.08). Internal reliability of the YPQ and its subscales was explored with Cronbach’s α coefficient. Test–retest reliability was also evaluated in the Control group by means of the intraclass correlation coefficient. Pearson correlation with RIBS was used to explore the convergent validity of the YPQ.

Regarding the effectiveness of the “*What’s up!”* intervention, to examine differences in primary and secondary outcome measures between the intervention and control groups, mixed-effects models (Gueorguieva and Krystal, 2004) were performed using Restricted Maximum Likelihood (REML) to estimate the parameters. One of the advantages of mixed models is that these methods account for the correlation between the repeated measurements (baseline, post-intervention, follow-up) for an individual. Since different high schools were assigned to study conditions, the statistical analyses were computed at the group level, where mixed effects models were used to account for random subject effects within each high school. Age, gender, and items concerning previous contact with people with a mental disorder, and having experienced a mental disorder were included as covariates. We tested the effect of a treatment group (intervention vs. control), time, and the interaction term (Group × Time). A separate model was estimated for each of the four outcome measures. Analyses were conducted separately for intervention completers and for the intent-to-treat (ITT) sample. We used multiple-imputation to impute the missing values.

CFA was conducted using the statistical software MPlus 7.4. For the other analyses, SPSS v.22 and STATA13.1 were used.

## Results

### Participants’ Characteristics

**FIGURE 1:**
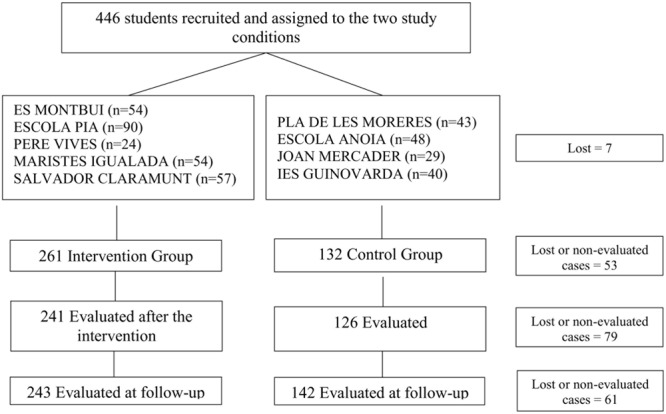
Flow chart of the “*What’s up!*” study; distribution of sample size by high school.

**Figure 1** shows the study flowchart. A total of 446 students were recruited and non-randomly assigned to the two study conditions. Their ages ranged from 14 to 17 years old (16 in the intervention group). Baseline evaluation was carried out in 393 students (88.12% of the initial sample); post-intervention evaluation in 367 students (82.29%) and follow-up was conducted in 385 students (86.32%). As these evaluations were carried out in the ordinary classrooms, absent students did not undergo the corresponding evaluation.

We did not find significant differences in baseline characteristics between study arms (see **Table 2**). Distribution by gender was very similar (52% women) and the mean age was approximately 14 years old. A fifth of the students (21%) had experienced some kind of mental health problem.

**Table 2:** Baseline characteristics of the sample by condition (Intervention and Control).

Variables		Total	Intervention	Control	*p-*Values
Gender (*n*, % women)		203 (51.7%)	137 (52.5%)	66 (50.0%)	0.67
Age (*M, SD*)		14.24 (0.47)	14.21 (0.43)	14.31 (0.54)	0.59
Own experience of a mental disorder (*n*, % Yes)		80 (20.6%)	46 (17.9%)	34 (25.8%)	0.09
Contact with mental disorders (*n*, % Yes)	A close relative	60 (15.4%)	42 (16.3%)	18 (13.7%)	0.56
	Another relative	87 (22.3%)	60 (23.3%)	27 (20.5%)	0.61
	A friend	164 (42.1%)	107 (41.5%)	57 (43.2%)	0.75
	An acquaintance	255 (65.2%)	172 (66.4%)	83 (62.9%)	0.50

No statistically significant differences were found between those students who answered at follow-up and those who did not for either the entire sample or both groups considered separately (all *p* > 0.05).

### Psychometric Analysis of the YPQ

CFA yielded significant χ^2^ values for both models (model 1 = 727.86, *p* < 0.01; model 2 = 434.99, *p* < 0.001). The other fit indices of model 2 indicated a more adequate fit to the data (CFI = 0.936; TLI = 0.924; RMSEA = 0.058, 90 and CI 0.051–0.065) than the first model (CFI = 0.863; TLI = 0.848; RMSEA = 0.082, 90 and CI 0.075–0.088). The reliability of the original version of the YPQ-SS was 0.79 and for YPQ-SAS it was 0.85 (Stuart et al., 2014).

In Model 2, the statistically significant correlated residuals were as follows: θ_14-19_= 0.29; θ_14-20_= 0.18; θ_14-21_= 0.23; θ_14-22_= 0.21; θ_19-20_= 0.53; θ_19-21_= 0.33; θ_20-21_= 0.57; θ_20-22_= 0.33; θ_21-22_= 0.36. A table with all the factor loadings can be found as Supplementary Material.

The overall internal consistency of the YPQ in our sample, based on Cronbach’s alpha among all items, was 0.84. We also calculated internal consistency at the scale level using the α “if item deleted” option. The alpha value for the total score ranged from 0.83 to 0.85. Regarding YPQ sub-scales, both present acceptable values (α = 0.73 for YPQ-SS and α = 0.8 for YPQ-SAS). Examining the α “if item deleted” option, the YPQ-SS and YPQ-SAS obtained values that ranged from 0.69 to 0.72; and 0.77 to 0.81, respectively. The test–retest reliability for the YPQ total score was 0.84, while the YPQ-SS and the YPQ-SAS both obtained 0.80. Convergent validity was also satisfactory as statistically significant correlations were found between the YPQ and its two factors, and the other measure used in this study (RIBS). The YPQ global score obtained correlations of *r* = 0.63 with RIBS; the YPQ-SS presented lower correlations, yet significant (*r* = 0.47). Correlations of *r* = 0.62 with RIBS were found for the YPQ-SAS.

### Effectiveness of the “What’s Up!” Intervention

**FIGURE 2:**
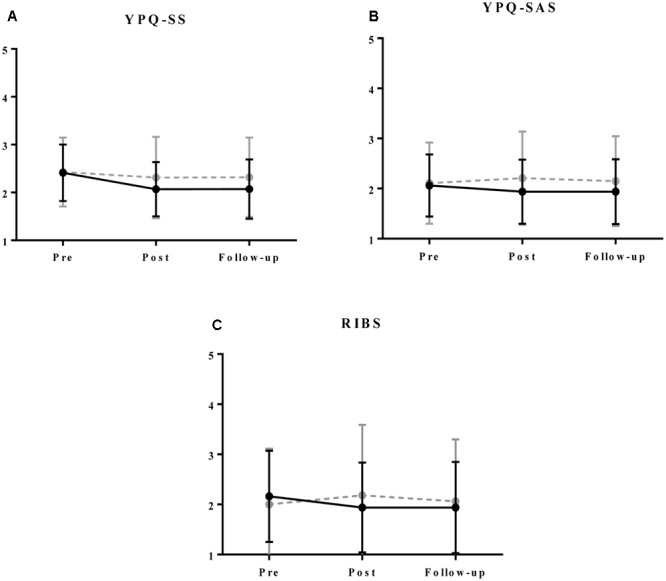
Mean scores and standard deviations for YPQ-SS **(A)**, YPQ-SAS **(B)**, and RIBS **(C)** by group throughout the study. Black and continuous line is for intervention group. Gray and dotted line is for control group. Higher scores in YPQ and RIBS represent higher stigma levels.

**Table 3** presents descriptive statistics for the study measures by group throughout the study. Statistical analyses revealed that the interaction Group (Intervention vs. Control) × Time (pre, post, and follow up) was significant for all measures (all *p* < 0.01). **Table 4** shows all the fixed and random effects of the models, which proved to be statistically significant for all measures (Chi squared: YPQ-SS = 259.42, *p* < 0.01; YPQ-SAS = 297.38, *p* < 0.01; RIBS = 217.02, *p* < 0.01). These interaction results imply that the groups differed in rate and manner of change over the course of the study. The YPQ sub-scale scores and RIBS scores in the intervention group improved significantly from baseline to post-intervention (*p* < 0.01 for YPQ-SS and *p* = 0.03 for YPQ-SAS; *p* = 0.01 for RIBS) and from baseline to follow-up (*p* < 0.01 for YPQ-SS and *p* = 0.01 for YPQ-SAS; *p* = 0.02 for RIBS). Interestingly, no statistically significant difference was found between the post-intervention evaluation and follow-up (all *p* > 0.05), which indicates that the changes observed after the intervention were maintained over time. In contrast, no differences were found from baseline to post-treatment in the control group (*p* = 0.06 for YPQ-SS, *p* = 0.16 for YPQ-SAS; *p* = 0.10 for RIBS) or between baseline and follow-up (*p* = 0.08 for YPQ-SS and *p* = 1.00 for YPQ-SAS; *p* = 1.00 for RIBS). As can be observed in **Figure 2** and **Table 3**, with the exception of the YPQ-SS, all measures worsened at the post-treatment evaluation and returned to baseline values at follow-up.

**Table 3:** Mean scores and standard deviations for the study measures (YPQ and RIBS).

	Intervention			Control		
	Baseline	Post	Follow-up	Baseline	Post	Follow-up
YPQ-SS	2.41 (0.59)	2.07 (0.57)	2.07 (0.62)	2.43 (0.72)	2.31 (0.85)	2.32 (0.83)
YPQ-SAS	2.06 (0.62)	1.94 (0.64)	1.94 (0.65)	2.11 (0.81)	2.21 (0.93)	2.15 (0.90)
RIBS	2.16 (0.91)	1.94 (0.90)	1.94 (0.91)	2.00 (1.11)	2.18 (1.41)	2.06 (1.24)

**Table 4:** Mixed effects models for YPQ-SS, YPQ-SAS, and RIBS (completers’ approach).

Parameter	YPQ-SS coefficient (*SE*)	YPQ-SAS coefficient (*SE*)	RIBS coefficient (*SE*)
**Fixed effects**			
Group	0.03 (0.08)	0.00 (0.11)	0.20 (0.12)
Time
T2	–0.10^∗^ (0.05)	0.10^∗^ (0.05)	0.18^∗^ (0.07)
T3	–0.09 (0.05)	0.06 (0.05)	0.08 (0.07)
Group × Time
G1 T2	–0.23^∗∗^ (0.06)	–0.21^∗∗^ (0.06)	–0.38^∗∗^ (0.09)
G1 T3	–0.23^∗∗^ (0.06)	–0.19^∗∗^ (0.06)	–0.28^∗∗^ (0.09)
Age	0.07 (0.05)	0.03 (0.05)	0.09 (0.07)
Gender (1 = woman)	–0.17^∗∗^ (0.05)	–0.33^∗∗^ (0.05)	–0.31^∗∗^ (0.07)
Close relative with a mental disorder (1 = yes)	–0.14^∗^ (0.07)	–0.08 (0.07)	–0.23^∗^ (0.10)
Other relative with a mental disorder (1 = yes)	–0.06 (0.06)	–0.06 (0.06)	–0.03 (0.08)
Friend with a mental disorder (1 = yes)	–0.10^∗^ (0.05)	–0.07 (0.05)	–0.17^∗^ (0.07)
Acquaintance with mental disorders (1 = yes)	–0.08 (0.05)	–0.05 (0.05)	–0.07 (0.07)
Personal experience of a mental disorder (1 = yes)	–0.11 (0.06)	–0.08 (0.07)	–0.23^∗^ (0.09)
**Random effects**			
High school	0.01 (0.01)	0.02 (0.01)	0.01 (0.01)
Students	0.12 (0.02)	0.15 (0.02)	0.28 (0.04)

*^∗^*p* < 0.05, ^∗∗^*p* < 0.01; G1 = Intervention group; T2 = Post-Intervention; T3 = Follow-up.*

Despite the significant interactions Group × Time, *post hoc* analysis revealed that groups did not differ significantly in any of the study periods in any of the study outcomes (all *p* > 0.05), which indicates that the change achieved by the intervention was small.

Analysis of covariates indicated that both stereotypical attitudes and intended behavior were significantly better in women than in men (all *p* < 0.01), and attitudes, measured with the YPQ-SS, were less stigmatizing in those students who had a close relative or a friend with mental health problems (*p* = 0.03; *p* = 0.04, respectively). Intended behavior measured with RIBS also presented significant effects of the covariates “having a close relative with a mental disorder” (*p* = 0.01), “having a friend with a mental disorder” (*p* = 0.01), and “having experienced mental disorders” (*p* = 0.01). The other covariates did not present significant effects for this scale.

Following multiple imputation of missing values (data available upon request), results were very similar to those obtained in the completers’ approach. Only some aspects changed: the effect of the follow-up measure became significant for the YPQ-SS (*p* < 0.01) but, surprisingly, the interaction Group × Time lost its significance at follow-up for the YPQ-SAS and the RIBS (*p* = 0.26 and *p* = 0.15, respectively). Furthermore, some of the covariates which presented no significant effect changed after the multiple imputation of missing values, such as age, which became significant for all the outcome measures (all *p* < 0.01).

## Discussion

The main objective of this study was to analyze the effectiveness of the “*What’s up!*” intervention on mental illness stigma in a large adolescent Catalan sample and, secondly, to examine, for the first time, the psychometric properties of the YPQ in a Catalan-speaking sample. The results can be summarized as follows: statistically significant but small improvements were found in the intervention group, and the psychometric analysis of the main outcome measure (YPQ) revealed a two-factor structure, good reliability coefficients for both dimensions (YPQ-SS and YPQ-SAS), and adequate convergent validity.

To our knowledge, this is the first study to report a full validation of the YPQ, as the original authors only assessed its internal reliability. A CFA was conducted, assuming that the two YPQ scales (i.e., YPQ-SS and YPQ-SAS) described by the authors would imply a two-factor structure. This analysis indicated that the YPQ shows a two-factor structure and similar psychometric properties to those reported by the designers of the measure (Stuart et al., 2014). A common result in CFA of psychological instruments composed of direct and reverse scored items is to obtain an inadequate fit because positively phrased items are prone to load on one factor and negatively phrased items on another (Woods, 2006). We decided to covariate the error terms of the reverse scored items to resolve this methodological problem and we obtained a better fit than without covariance. The YPQ had good internal consistency and convergent validity with the RIBS. When taking the questionnaires’ two scales separately, both correlated significantly with the RIBS. All items loaded correctly on their respective factor with the exception of item 4 (“People with a mental illness could snap out of it if they wanted to”), which presented a very low λ for both models.

Without ignoring that our sample may not reflect the whole variability of adolescence as its age variability is very low, our results show that the YPQ is a valuable instrument for use in further studies addressed to secondary students. Its value lies not only in its sound psychometric properties but also in the adequacy of the language and the aspects evaluated, which are directly related to the reality of adolescence (e.g., sitting next to a classmate with a mental disorder or being taught by a teacher with mental health problems). This favors the YPQ in comparison with other commonly used measures such as the Community Attitudes toward Mental Illness (CAMI; Taylor and Dear, 1981), whose items contain questions related to the health system or the economic costs of people with a mental disorder, areas which are not often well-known by teenagers. Moreover, the YPQ items reflect the realities of today’s teenager, such as bullying or volunteering. Many measures were designed for previous studies, but not all of them were reliable and valid (Sakellari et al., 2011). Consequently, the present study provides validation of the YPQ to help overcome the difficulty of choosing which measure to use when delivering an anti-stigma intervention to a teenage population.

Regarding the effects of the intervention, we can conclude that *“What’s up!”* was able to improve the attitudes and intended behavior of the students. Despite being significant, the changes achieved by the intervention were small, similar to those observed in some previous studies (Mino et al., 2001; Altindag et al., 2006; Kerby et al., 2008; Lincoln et al., 2008), although the reason why these interventions did not have a bigger impact was considered to be the fact that they were addressed to medical students, a population with such high levels of knowledge about mental disorders that they focused more on the negative aspects of these conditions, making their stigma stronger (Corrigan and Watson, 2007; Angermeyer et al., 2011). Nonetheless, this cannot be considered the reason why *“What’s up!”* has not presented more significant effects on reducing stigma, as our sample was constituted of high school students who did not have such a degree of knowledge. In all likelihood, the fact that the students already presented very low levels of stigma before the intervention (floor effect) was in part responsible for the small effects of the intervention. This is in line with previous findings showing the Spanish population reporting lower levels of stigma than those of other European and American countries (Aznar-Lou et al., 2016), which would imply that it could be more efficient to address interventions such as “*What’s up!”* to students who report high levels of stigma.

To our knowledge, this is the first time that the effectiveness of a curricular intervention addressed to fight stigma toward mental disorders has been explored. Previous approaches were commonly based on short but very immersive workshops which used materials such as presentations, videos, role-plays, talks, and contact with people with mental disorders. Some were reported to have achieved a greater impact on stigma than “*What’s up!”* (Petchers et al., 1988; Mound and Butterill, 1993; Esters et al., 1998; Rahman et al., 1998; Ng and Chan, 2002; Pinfold et al., 2003; Schulze et al., 2003; Watson et al., 2004; Stuart, 2006; Spagnolo et al., 2008; Essler et al., 2009), mostly on knowledge about mental health problems (Sakellari et al., 2011), an aspect that was not evaluated in our study. However, many of these studies had methodological shortcomings such as the absence of a control group (Mound and Butterill, 1993; Pinfold et al., 2003; Stuart, 2006; Essler et al., 2009) and only three conducted a follow-up, so these results should be considered with caution. Additionally, a very common limitation of previous interventions was that they seldom reported sustained improvements over time (Economou et al., 2012; Thornicroft et al., 2016), which implied that they had short-term effects that were not maintained at follow-up. In contrast, our results indicate that the improvements in stigmatizing attitudes and intended behavior achieved by “*What’s up!”* were maintained at 9-month follow-up. It is possible that the model of intervention used for this campaign, where the teachers include the anti-stigma material in their classes, makes it easier for the students to get involved with the information than in those interventions in which an external professional brings the material to the adolescents in a workshop format. This may produce a greater immediate reduction in stigma as it is more immersive than doing their regular classes with some didactic units on mental health, but its effects may be more superficial and easily lost with the passage of time.

These results suggest that the debate on which approach is more effective to fight stigma is open. The literature provides different examples of interventions addressing this issue (workshops, whole-school approaches, and now, curricular interventions). Considering the characteristics of the target population and the available resources to conduct the intervention, the most adequate alternative is chosen. In the case of curricular interventions such as *“What’s up!,”* our results suggest that they can be effective in promoting sustained effects regarding stigmatizing attitudes and intended behavior toward people with mental disorders. These improvements have been found in a sample with relatively low levels of stigma, which may imply that other populations with similar levels may benefit from the intervention as well. Since the results of “*What’s up!”* were maintained after a 9-month follow-up, it may be also considered as an affordable extension to other approaches that have already proved to be effective but only in the short-term.

In another vein, it is also interesting to discuss the significant influence of the covariates in the effectiveness findings. For instance, being female was a significant covariate for all the measures, which is in line with previous studies (Stickney et al., 2012). Some studies have found that adolescent girls had more knowledge and experience about mental disorders, reported less stigma toward people with them and felt more likely to use mental health services (Barrett and Raskin White, 2003). The statistical analysis did not find a significant effect of being female on the response to the intervention, but this effect has been found in previous studies (Thornicroft et al., 2007). A possible explanation for this could be that traditional ideals of masculinity (e.g., success, power, competition, emotional repression) influence teenage boys in such a way that messages designed to combat stigma with respect to mental disorders have a smaller impact than they do on teenage girls (Chandra and Minkovitz, 2006).

Two other covariates that also appeared to be significant in all the measures but the YPQ-SAS were having a close relative with a mental disorder and having a friend with a mental disorder. Again, no significant effect of these variables on the response to the intervention was found, so it cannot be concluded that these adolescents are more likely to decrease their stigma. However, it can be asserted that their levels of stigma were significantly lower than those reported by the teenagers who did not have friends or close relatives with a mental disorder. It may be the case that having a close relationship with someone with a mental disorder makes it easier for teenagers to feel empathy with them and so to decrease their levels of stigma more significantly than adolescents who have no friends or close relatives with this kind of problem, as it has been observed that empathy is a key individual factor influencing attitude change toward mental disorders (Couture and Penn, 2003).

### Strengths and Limitations

To our knowledge, this is the first study to report the effectiveness of a curricular intervention to combat stigma associated with mental disorders. This is a controlled study, but not randomized, so there could be some selection bias. In this respect, it is notable that the comparative analyses in the basal phase showed no significant differences between the intervention and the control group in any of the study measures. Due to the low number of independent variables of this study, no mediation analysis was conducted.

Some limitations about the intervention itself must be considered: on the one hand, the instructions given to the teachers were very flexible, requiring the application of just three of the nine didactic units proposed by the project, although remarking that the first and the last (which contained the social contact component of the campaign) were considered “essential.” It can be concluded that a little more systematic orientation in applying the intervention is needed to evaluate intervention effectiveness, although it should be noted that all high schools implemented at least five of the nine didactic units and an important strength of the intervention was that this degree of flexibility made *“What’s up!”* a truly implementable intervention in the real context of high schools.

We must take into account that the timing was not the same for all the high schools at the post-intervention measure. Although all started within 2 weeks of each other, one class took the post-intervention measure only 25 days after baseline assessment, while the rest of the classes were evaluated between 77 and 105 days after baseline. These differences may have had an impact on the results as it seems reasonable to suppose that the more time spent on didactic units in class, the more impact they would have on the students. This could be easily corrected by adjusting the evaluation periods and conducting assessments at roughly the same time in all schools. Additionally, during application of the intervention there was no register of external events that could interfere with it (e.g., to include an item in the socio-demographic scale registering contact with fictional characters with mental health problems either in movies, TV shows, or novels).

Regarding the external validity of the intervention, it should be borne in mind that, although the measures used for this study presented strong psychometric properties, their items might be susceptible to social desirability and more objective measures should be developed to ensure that the assessment of stigma is as close to actual behavior as possible. It would also have been interesting to include a qualitative measure in the evaluation of the impact of the intervention to determine which aspects had the biggest effect on the participants and why. This could have been especially useful considering that “*What’s up!”* consisted of nine distinct units implemented by different teachers, which, as mentioned previously, introduced a high degree of variation to the intervention, and not all the units could impact equally for every student. Future studies with a larger sample size could use a “dismantling design” to examine which of the multiple didactic units included in the “*What’s up!*” intervention are the active ingredients of change. One typical method for conducting this type of study is to compare single intervention components with the global intervention.

As noted above, an important strength of this study is the use of reliable and valid outcome measures. The YPQ emerged as a useful instrument that measures both attitudes and intended behavior and whose psychometric attributes have been found to be positive. However, it is true that its convergent validity was only studied with RIBS, which is an intended behavior measure, and it would also be interesting to study it with a measure of stigmatizing attitudes such as the CAMI. It could also be argued that this measure is not very sensitive to change, but as similar results were found for RIBS, we must conclude that the intervention did not have the impact required to produce more significant changes, maybe due to some of its previously explained limitations. Moreover, we should point out that our results may not be totally generalizable to the whole adolescent population, as our sample presented a very low degree of age variability.

## Conclusion

*“What’s up!”* is a promising, original intervention model in which teachers are the responsible for introducing campaign materials into regular didactic units, without modifying the school curriculum and offering the students the opportunity to learn about mental health through education and social contact. Although significant improvements were observed, these results were small. To overcome the shortcomings of this intervention, further anti-stigma interventions could focus on reducing time differences between classes and registering external variables that could affect students’ stigma levels. Additionally, it may be more appropriate to address the intervention especially to those students who report higher levels of stigma at baseline to counter the “floor effect.”

On the other hand, the present work has satisfactorily validated the YPQ, an instrument that had only been used in one previous intervention and whose psychometric properties had not yet been reported. This questionnaire evaluates stereotypical attitudes and intended behavior and presents good internal consistency, test–retest reliability and convergent validity and can thus be considered as a valuable measurement instrument for use in further studies of stigma in adolescent populations.

## Author Contributions

AP-A and LA-R made substantial contribution to the analysis and to the interpretation of the data, drafted the manuscript, provided final approval of the version to be published, and agreed to be accountable for all aspects of the work in ensuring that questions related to the accuracy or integrity of any part of the work are appropriately investigated and resolved. JL, IA-L, MR-V, and AF-S made substantial contributions to the conception and the design of the study, drafted the manuscript, provided final approval of the version to be published, and agreed to be accountable for all aspects of the work in ensuring that questions related to the accuracy or integrity of any part of the work are appropriately investigated and resolved. AS-B helped out in the interpretation of data for the work, revised the manuscript critically for important intellectual content, provided final approval of the version to be published, and agreed to be accountable for all aspects of the work in ensuring that questions related to the accuracy or integrity of any part of the work are appropriately investigated and resolved. MJ and AT revised the manuscript critically for important intellectual content, provided final approval of the version to be published, and agreed to be accountable for all aspects of the work in ensuring that questions related to the accuracy or integrity of any part of the work are appropriately investigated and resolved. AB revised the manuscript critically for important intellectual content, provided final approval of the version to be published, and agreed to be accountable for all aspects of the work in ensuring that questions related to the accuracy or integrity of any part of the work are appropriately investigated and resolved.

## Conflict of Interest Statement

The authors declare that the research was conducted in the absence of any commercial or financial relationships that could be construed as a potential conflict of interest.
