# Supplementary material for: Effectiveness of the “What’s Up!” Intervention to Reduce Stigma and Psychometric Properties of the Youth Program Questionnaire (YPQ): Results from a Cluster Non-randomized Controlled Trial Conducted in Catalan High Schools
Source: Front Psychol. 2017 Sep 14;8:1608. doi: 10.3389/fpsyg.2017.01608 (PMC5603659; doi:10.3389/fpsyg.2017.01608)
Supplement: Supplementary file 1 [file Table_1.DOCX]

**Supplementary Table 1.** Means (M), standard deviations (SD), and factor loadings (λ) for the Catalan version of YPQ items in the CFA

| **Item** | **M (SD)** | **Model 1** | | **Model 2** | |
| --- | --- | --- | --- | --- | --- |
|  |  | **λ F1** | **λ F2** | **λ F1** | **λ F2** |
| 1. Most people with a mental illness are too disabled to work. | 2.32 (.95) | .49 | - | .48 | - |
| 2. People with a mental illness tend to bring it on themselves. | 2.39 (1.01) | .38 | - | .38 | - |
| 3. People with mental illnesses often don’t try hard enough to get better | 2.15 (1.00) | .39 | - | .39 | - |
| 4. People with a mental illness could snap out of it if they wanted to. | 3.12 (2.41) | .15 | - | .16 | - |
| 5. People with a mental illness are often more dangerous than the average person. | 2.41 (1.12) | .70 | - | .69 | - |
| 6. People with a mental illness often become violent if not treated. | 2.79 (.94) | .51 | - | .52 | - |
| 7. Most violent crimes are committed by people with a mental illness. | 2.31 (1.14) | .47 | - | .47 | - |
| 8. You can’t rely on someone with a mental illness. | 1.93 (.89) | .74 | - | .74 | - |
| 9. You can never know what someone with a mental illness is going to do. | 3.41 (1.08) | .49 | - | .49 | - |
| 10. Most people with a mental illness get what they deserve. | 1.28 (0.68) | .59 | - | .59 | - |
| 11. People with mental illnesses need to be locked away. | 2.38 (1.28) | .56 | - | .56 | - |
| 12. I would be upset if someone with a mental illness always sat next to me in class. | 1.89 (1.00) | - | .76 | - | .79 |
| 13. I would not be close friends with someone I knew had a mental illness. | 2.16 (1.05) | - | .76 | - | .78 |
| 14. I would visit a classmate in hospital if they had a mental illness. | 2.03 (.98) | - | .52 | - | .45 |
| 15. I would try to avoid someone with a mental illness. | 2.08 (.96) | - | .77 | - | .78 |
| 16. I would not mind it if someone with a mental illness lived next door to me. | 2.07 (1.15) | - | .40 | - | .40 |
| 17. If I knew someone had a mental illness I would not date them. | 2.70 (1.15) | - | .65 | - | .67 |
| 18. I would not want to be taught by a teacher who had been treated for a mental illness. | 2.45 (1.26) | - | .56 | - | .58 |
| 19. I would tell a teacher if a student was being bullied because of their mental illness. | 1.61 (.88) | - | .48 | - | .27 |
| 20. I would stick up for someone who had a mental illness if they were being teased. | 1.57 (.76) | - | .64 | - | .43 |
| 21. I would tutor a classmate who got behind in their studies because of their mental illness. | 1.91 (.88) | - | .59 | - | .44 |
| 22. I would volunteer my time to work in a program for people with a mental illness. | 2.41 (1.04) | - | .51 | - | .43 |

*Note*: Model 1 corresponds to the distribution of items in the original version of the scale (YPQ-SS and YPQ-SAS subscales), model 2 is a respecification of model 1 including method effects. F1 = Stereotype Scale (YPQ-SS); F2= Social Acceptance Scale (YPQ-SAS)
